# DNA Compaction Induced by a Cationic Polymer or Surfactant Impact Gene Expression and DNA Degradation

**DOI:** 10.1371/journal.pone.0092692

**Published:** 2014-03-26

**Authors:** Marie-Louise Ainalem, Andrew Bartles, Joscha Muck, Rita S. Dias, Anna M. Carnerup, Daniele Zink, Tommy Nylander

**Affiliations:** 1 Physical Chemistry, Center for Chemistry and Chemical Engineering, Lund University, Lund, Sweden; 2 Ludwig-Maximilians-Universität München, Department Biologie II, Planegg-Martinsried, Germany; University of Quebect at Trois-Rivieres, Canada

## Abstract

There is an increasing interest in achieving gene regulation in biotechnological and biomedical applications by using synthetic DNA-binding agents. Most studies have so far focused on synthetic sequence-specific DNA-binding agents. Such approaches are relatively complicated and cost intensive and their level of sophistication is not always required, in particular for biotechnological application. Our study is inspired by *in vivo* data that suggest that DNA compaction might contribute to gene regulation. This study exploits the potential of using synthetic DNA compacting agents that are not sequence-specific to achieve gene regulation for *in vitro* systems. The semi-synthetic *in vitro* system we use include common cationic DNA-compacting agents, poly(amido amine) (PAMAM) dendrimers and the surfactant hexadecyltrimethylammonium bromide (CTAB), which we apply to linearized plasmid DNA encoding for the luciferase reporter gene. We show that complexing the DNA with either of the cationic agents leads to gene expression inhibition in a manner that depends on the extent of compaction. This is demonstrated by using a coupled *in vitro* transcription-translation system. We show that compaction can also protect DNA against degradation in a dose-dependent manner. Furthermore, our study shows that these effects are reversible and DNA can be released from the complexes. Release of DNA leads to restoration of gene expression and makes the DNA susceptible to degradation by Dnase. A highly charged polyelectrolyte, heparin, is needed to release DNA from dendrimers, while DNA complexed with CTAB dissociates with the non-ionic surfactant C_12_E_5_. Our results demonstrate the relation between DNA compaction by non-specific DNA-binding agents and gene expression and gene regulation can be achieved in vitro systems in a reliable dose-dependent and reversible manner.

## Introduction

In the cell nucleus DNA is complexed with positively charged histone octamers, giving rise to the nucleosome structure. The nucleosome structure plays an important role in transcriptional regulation [1]. In addition the patterns of histone modifications, giving rise to the so-called histone code, are important for transcriptional regulation [2]–[6]. Acetylation of specific lysine residues, which reduces the number of normally positively charged sites, is associated with chromatin decondensation and transcriptional activity [7]–[10]. Chromatin decondensation, which is often microscopically visible or can be detected at the molecular level as an increase in the sensitivity to DNA degradation by nucleases [10], appears to affect not only the nucleosome structure, but also higher levels of chromatin organization, which so far has not been characterized in detail. Although the degree of chromatin compaction at nucleosomal and higher-order levels usually correlates with its transcriptional activity, many exceptions have been observed. In fact also relatively condensed heterochromatin can show transcriptional activity under certain condition [11]. However, it is still unclear to what extent and how chromatin compaction alone, or together with sequence specific silencing mechanisms, contribute to gene regulation *in vivo*.

There is also great interest in achieving gene regulation by using synthetic compounds for in vitro systems, *e.g.* in biotechnological applications. Sequence specific DNA-binding synthetic agents such as triplex-forming oligonucleotides [12]–[16], zinc-finger proteins [17]–[19], peptide nucleic acids [20]–[24], and synthetic polyamides [25], [26] have been used as gene silencers. However, these approaches rely on the base specificity of the promoter region for the binding of the agents that inhibit transcription, interfering with either the initiation of transcription or elongation. This strategy is challenging to implement in practice because the appropriate target DNA sequence must be identified. Furthermore, for many applications, such as large-scale protein production, this level of sophistication is too costly and not even necessary. Here, non-specific DNA-binding agents are of large interest, but not fully exploited.

Non-specific DNA-binding agents have been used in studies aiming to develop non-viral gene delivery vehicles. These studies have also been inspired by recent developments in bionanotechnology and facilitated by new experimental and synthetic tools that came with these developments. In particular dendrimers [3], [6], [27]–[31], surfactants/lipids [32]–[34], and polyamines [7], [35], [36] are known to interact electrostatically with DNA. These agents have also been shown to be able to regulate DNA transcription [6], [32]–[34]. The challenge here is to control the nanostructure of the formed complexes.

Dendrimers can be made highly cationic and are, unlike most commercial cationic polymers, monodisperse in both size and charge. The most studied specimen is the poly(amido amine) (PAMAM) dendrimer which is the dendrimer type used in this study. Specifically PAMAM dendrimers of generation 4 (G4), which have 64 primary amine functional surface groups and are similar in size and charge as the histone octamer [37], [38], was one of the two non-specific DNA-binding agents applied in this study.

Cationic surfactants also form complexes with DNA and the interactions between the surfactant and the DNA have therefore been subject to a number of studies [39]–[41]. The polar headgroup of the most common cationic surfactants is only monovalent and individual surfactant molecules are therefore not sufficient to induce DNA compaction. However, due to their amphiphilic characteristics, surfactants self-assemble into complexes of nanoscopic dimensions in the vicinity of DNA, which in turn leads to the compaction of DNA. As the self-assembly of surfactants is relatively easy to control, it is in principle possible to regulate the compaction of DNA in this way. In fact, this concept has been used to improve the efficiency of other positively charged agents, e.g., hydrophobically modified spermidine [8], [42], [43] and amphiphilic peptides (lipopeptides) [21], [44]–[46]. Upon further addition of surfactant, the compacted DNA molecules will aggregate and precipitate as complexes that can have a highly organized structure. The second non-specific DNA-binding agent selected for use in this study was the cationic surfactant hexadecyltrimethylammonium bromide (CTAB). In particular, DNA and CTAB form a normal hexagonal phase [47].

The challenge for biophysicists is to establish the relationship between a certain nano-scale structure and the biomolecular activity. One very good example, which served as an inspiration for the present study, is the work of Bielinska et al, where DNA–dendrimer complexes were used to transfer oligonucleotides and plasmid DNA into *in vitro* cell culture system [48]. Moreover it was found that this could be a way to transfect luciferase antisense expression plasmid and thus a way to regulate the expression of luciferase. Our study is much less sofisticated in the sense that it uses cell-free systems. Hower, it allows us to directly compare the effect of compaction on the accessibility of the DNA. Athough we are far from the biological system, the same mechanisms should apply, although with less parameters to modulate. Another motivation for this study is the growing interest in cell free protein synthesis as described in a recent review [49]. Here the ability to regulate transcription in *in vitro* systems is important. The first objective of the present study is to relate the compaction of DNA using a cationic polymer or surfactant to 1.) the accessibility of DNA for Dnase catalysed degradation and 2.) gene expression under similar conditions. Here we will compare the effect of using a compacting agent that is multivalent (G4 dendrimer) with a monovalent surfactant that assemblies into a multivalent one, i.e. a surfactant micelle. The second objective is to exploit the possibilities to reverse the compaction for the two types of compacting agents and restore the transcription capability. This is essential for applications such as the pretreatment of the samples for Diagnostic PCR and forensic DNA analysis [50]. For decompaction we used a highly negatively charged polyelectrolyte, heparin as well as an anionic, SDS, and a non-ionic surfactant, C_12_E_5_.

## Materials and Methods

### Sample preparation

Luciferase plasmid DNA (Promega, 4331 basepairs (bp)), was amplified, linearized and purified as described in detail elsewhere [27]. Stock solutions were prepared using 10 mM NaBr. G4 PAMAM dendrimers were purchased from Sigma (lot no 412449) as 10 wt% solutions in methanol. Before use, the methanol was evaporated under reduced pressure at room temperature and the dendrimers were resolubilized in aqueous solutions of 10 mM NaBr (Aldrich). Stock solutions of DNA and G4 dendrimers were stored at 4°C. The cationic surfactant CTAB was obtained from Merck, and recrystallized three times with an acetone/ethanol mixture before use. To prepare the CTAB stock solutions the desired surfactant amount was weighed and dissolved in 10 mM NaBr.

Samples were prepared by adding dendrimer or surfactant solutions (of varying concentration) into equal volumes of a DNA solution of the desired concentration in 10 mM NaBr prepared from Milli-Q purified water (specific resistivity of 18.2 M Ωcm). All dendrimer-containing samples were left on mixing boards at 25°C for 3 h before analyzed, whereas CTAB-containing samples were equilibrated for at least 1 h at 25°C before analyzed. Results are presented as a function of the charge ratio, (*r*_charge_), defined as the ratio between the charged groups on the dendrimer (NH_3_^+^) or surfactant (N(CH_3_)_3_^+^) and the DNA nucleotides (PO_4_^−^). We have chosen to use *r_charge_* and not concentration ratio to be able to directly compare the results for the multivalent dendrimers with those of the monovalent surfactant. It was also assumed that all primary amine groups are protonated under the conditions used [51]–[53].

### The association between DNA and compacting agents

Steady state fluorescence spectroscopy (2 μg mL^−1^ DNA) and gel electrophoresis (25 μg mL^−1^ DNA) were used to investigate the accessibility of DNA molecules to small fluorophore molecules (GelStar and ethidium bromide (EtBr)). DNA compaction using CTAB and G4 were, in addition, visualized using cryo-TEM (0.1 mg mL^−1^ DNA). For the fluorescence spectroscopy measurements, a Cary Eclipse fluorescence spectrometer (Varian) on 384-well plates was used for the CTAB system. For the G4 experiment, a Perkin-Elmer LS-50B spectrometer using a 10×10 mm quartz cuvette from Hellma was utilized. Excitation and emission slits were chosen to be 5.0 nm. The fluorophore used for DNA (2 μg mL^−1^) was the GelStar nucleic acid stain (Cambrex), which has an excitation maximum (*λ*_ex_) of 493 nm and an emission maximum (*λ*_em_) of 527 nm. To optimize measurement quality, the 10,000× concentrated stock solution of GelStar was diluted to 10× for the plate reader experiments and 2.5× for the Perkin-Elmer spectrometer. Samples were equilibrated for at least 30 min in the presence of GelStar before the measurements were started. The gel electrophoresis experiments were performed using 1 wt% of Seakem LE Agarose (Cambrex) and 25 μg mL^−1^ DNA. The gels were either pre-stained with GelStar or post-stained using EtBr. The samples for cryo-TEM were prepared using a controlled environment vitrification system (CEVS) [54], in accordance with [27]. Transmission electron micrographs were digitally recorded using a Philips CM120 Bio TWIN electron microscope, operated at 80 kV, equipped with a Gatan MSC791 cooled-CCD camera system. To minimize beam damage, all samples were imaged under minimal electron dose conditions.

### *In vitro* transcription using T7 polymerase

Luciferase synthesis was investigated using a Megascript kit (Promega) for samples containing 10 μg mL−1 DNA. Samples containing compacted DNA formed in aqueous solutions of 10 mM NaBr (as described above), were transferred to aqueous solutions containing a reaction buffer required for in vitro transcription (tRB). The tRB contained 40 mM Trizma, 10 mM DTT, and 0.01% (v/v) Triton X-100 in addition to di- and trivalent species that are needed for the transcription to work but are also known to promote DNA compaction; 25 mM MgCl_2_ and 2.5 mM spermidine. The assembled reaction solutions were incubated at 37°C for 2 h and both gel electrophoresis and quantitative analyses using a Cary Eclipse plate reader were used to verify transcription inhibition.

#### Gel electrophoresis

Sample mixtures were loaded onto a precast RNA gel (Cambrex) and the RNA gels were post-stained using GelStar in ammonium acetate buffer and imaged using transillumination.

#### Quantitative analysis using the plate reader

After incubation, samples were heated to 65°C to inactivate the polymerase before RNA was quantified using the SYTO RNASelect green fluorescent cell stain (Invitrogen) with *λ*_ex_ = 490 nm and *λ*_em_ = 530 nm. A final concentration of 0.5 μM was used and samples were let to equilibrate for at least 30 min before measurements. The stock solutions of the fluorophore were protected from light and stored at 4°C.

### *In vitro* translation using T7 polymerase

A coupled cell free transcription-translation system (TNT Coupled Reticulocyte Lysate System, Promega L4610) was used to synthesize luciferase for the G4/DNA and the CTAB/DNA system. Samples containing compacted DNA, formed in aqueous solutions of 10 mM NaBr (as described above), were transferred to the tRB (see above for the components included). Reactions containing 10 μg mL^−1^ DNA were incubated for 90 min at 30°C and the produced luciferase was detected by the addition of 50 μL luciferase assay reagent (Promega) to 5 μL of sample using a FluoStar Optima chemiluminometer (BMG labtech) containing a Polarstar Optima illuminator. Light intensity measurements were started 5 min after the addition of the assay reagent and only the initial value was considered. All samples were measured twice.

### Dissociation of DNA from complexes

To samples containing G4/DNA and CTAB/DNA complexes, a varying concentration of co-solutes was added. Sodium dodecyl sulphate (SDS, Fluka), C_12_E_5_ (Fluka) and heparin sodium salt from porcine intestinal mucosa (Sigma Aldrich) were added to the G4/DNA complexes, respectively, and let to equilibrate for 30 min before evaluation. To the CTAB/DNA complexes we added C_12_E_5_ (Fluka) and samples were equilibrated for 1 h. Evaluation was performed using the Cary Eclipse plate reader (2 μg mL^−1^ DNA) and gel electrophoresis (25 μg mL^−1^ DNA).

### Degradation of DNA

Dnase I (Turbo Dnase I, Ambion) was used to elucidate how the degree of compaction affects the protection against enzymatic digestion of DNA. 1 unit of Dnase I (which, under ideal conditions in the presence of the Turbo Dnase I reaction buffer and at 37°C, degrades 1 μg of DNA in 10 min) was added to each DNA-compacting agent mixture. The study was performed using 10 mM NaBr in the absence of the Turbo Dnase I reaction buffer. Samples were incubated at 37°C for at least 20 min to ensure full digestion of the accessible parts of the DNA molecules. After incubation with Dnase I, G4/DNA samples were heated to 70°C for 15 min to ensure enzymatic inactivation. Heparin (10 μg mL^−1^) was then added to release DNA from G4. Samples were equilibrated for 30 min and evaluated using gel electrophoresis.

## Results and Discussion

### The interaction between DNA and compacting agent – a quantitative estimation

DNA undergoes a conformational transition from a semi-flexible coil to a more compacted state upon mixing with PAMAM dendrimers or CTAB surfactants. Dynamic light scattering and cryogenic transmission electron microscopy (cryo-TEM) studies have previously shown that the complex formation between DNA and G4 dendrimers is a cooperative process [27], [30], [55]. This also applies to cationic surfactants [56] (*e.g.*, CTAB) and many other compacting agents, such as polyamines [41]. Cooperative DNA compaction results in the coexistence of free DNA, adopting a random coil conformation, and compacted DNA molecules [40]. The reason for this cooperative binding is the strong attractive ion correlation effect resulting in a correlated positioning of the counterions. Further binding by the compacting agent on the partially compacted DNA is therefore preferred relative to the free DNA molecules. However, it is important to bear in mind that the fact that a cooperative process occurs does not necessary imply that the formed aggregates are uniform. In fact for low charge ratios, *r*_charge_ <1, G4 dendrimers induce a mixture of rods, toroids and globular complexes [6], [27], [57]. We have also previously observed that significant morphological rearrangement occurs for DNA compacted with the lower generation (1–2) dendrimers which with time leads to the formation of toroidal complexes [3]. This does not occur for higher generation dendrimers, possessing high charge density, where the dendrimers are thought to be kinetically trapped as soon as they bind to the DNA strand and the resulting morphology is a less well-defined globular one [27].

**Figure 1:**
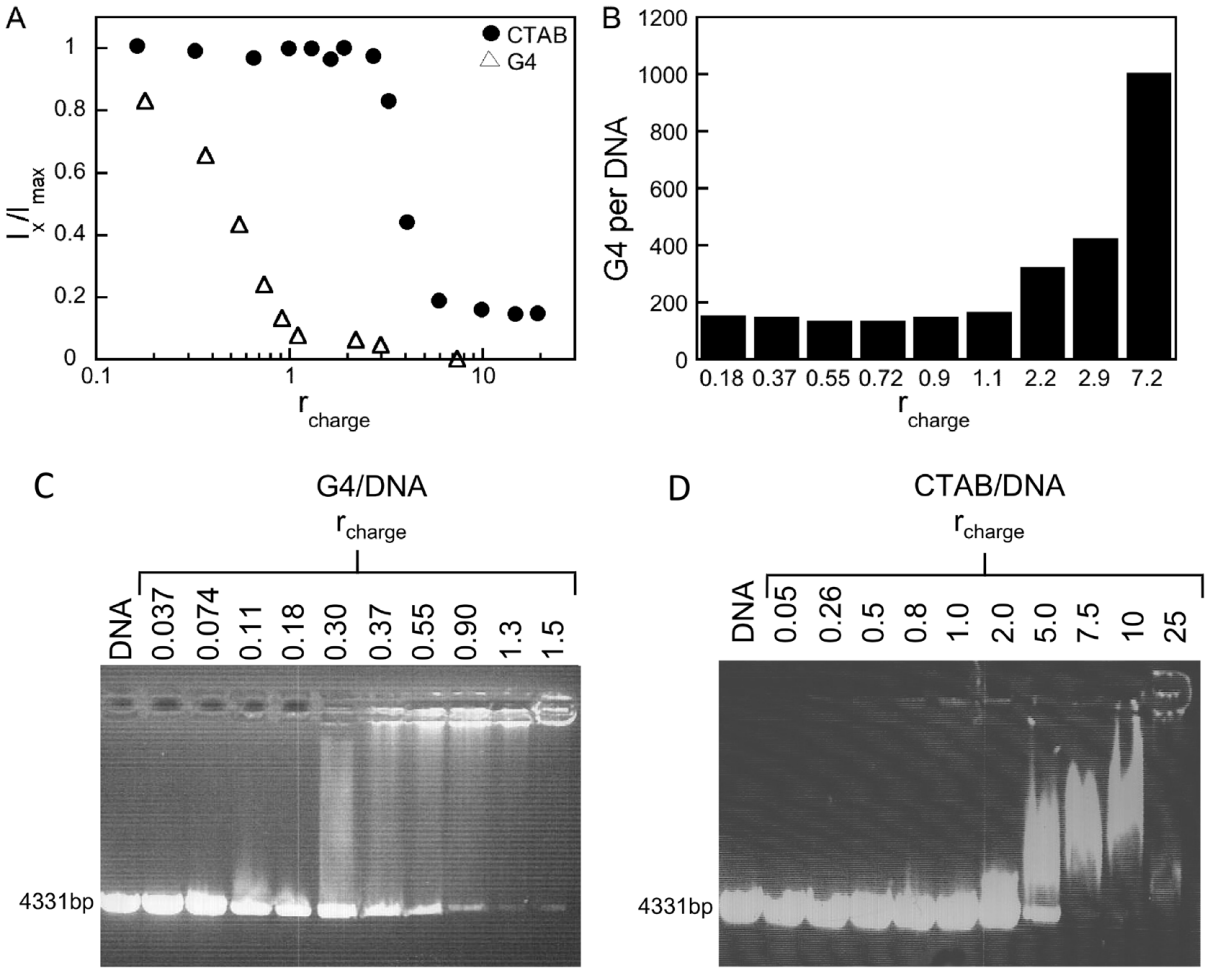
DNA condensation using G4 dendrimers and CTAB surfactants. (A) The fluorescence intensity of GelStar bound to DNA, shown as a function of *r*_charge_ in solutions containing 10 mM NaBr for G4 dendrimers (▵) and CTAB (•). Data are normalized to the amounts produced in the samples only containing DNA (in the absence of dendrimer or surfactant) and the *I*_max_ value is linearly dependent on the amount of DNA that is available to bind GelStar. The DNA concentration is 2 μg mL^−1^ and error bars are smaller or equal to the size of the markers. (B) The mean number of G4 per compacted DNA chain at varying *r*_charge_, calculated as described in the text. Note that below charge neutralization the amount of bound G4 per DNA strand is constant, that is each complex contains the same number of G4. Once the neutralization point is reached, the solution only contains compacted DNA and the number of G4 per compacted DNA increases. The results from the electrophoreses study - DNA condensation by G4 dendrimers and CTAB surfactants - are shown in (C) (D), respectively. Lane 1 in both C and D displays free linearized plasmid DNA in the absence of any compacting agent (control, 4331 bp). Samples in lanes 2–11 contain increasing amounts of the compacting agent and the corresponding *r*_charge_ values are indicated. The DNA concentration was 25 μg mL^−1^ and the gels were stained with Ethidium Bromide (EtBr).

**Figure 2:**
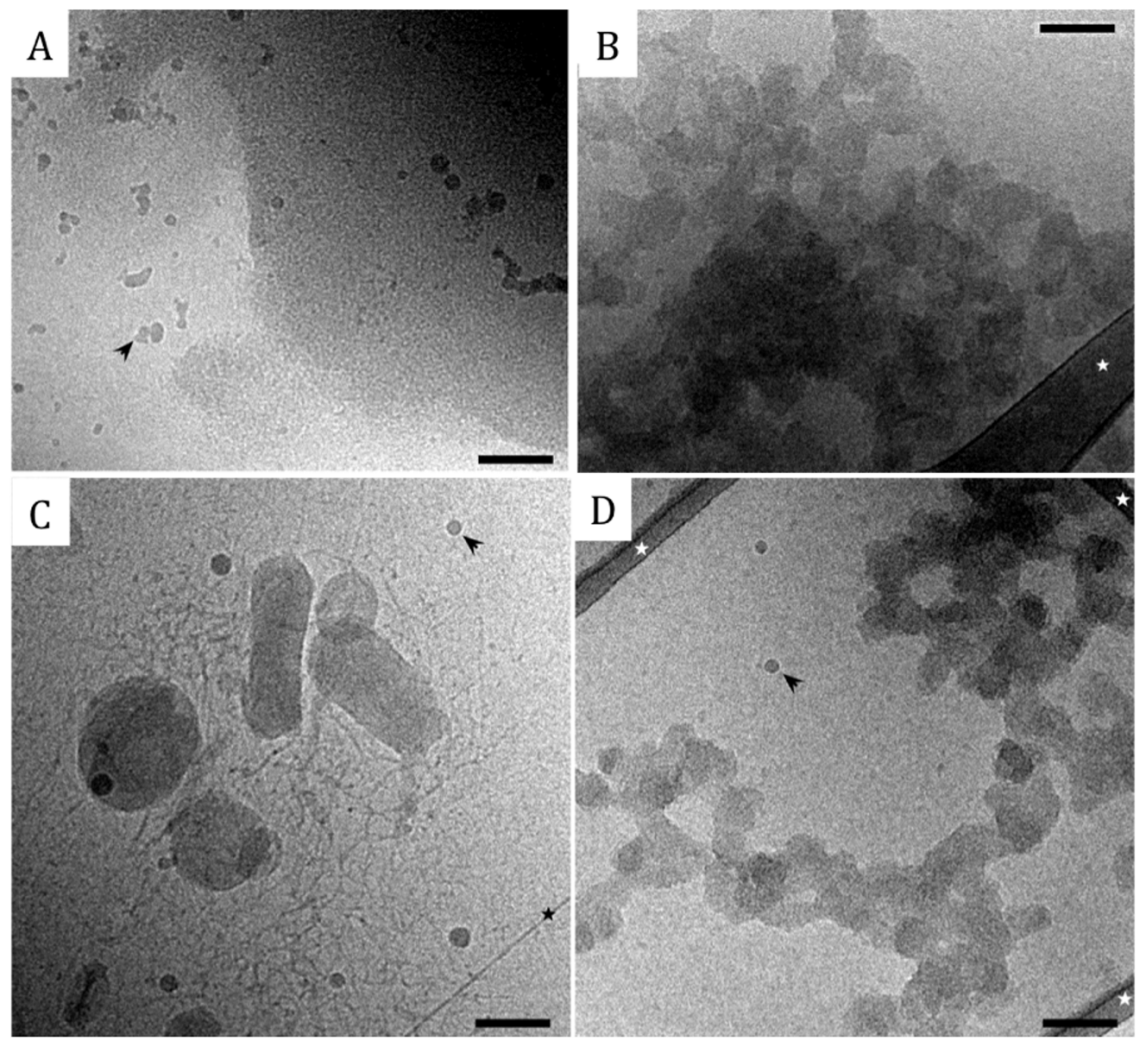
Cryo-TEM images of DNA (0.1 mg mL^−1^) complexes. (A, C) G4/DNA of *r*_charge_  = 0.5, and (B, D) CTAB/DNA of *r*_charge_  = 7.5. All samples were prepared in aqueous solutions of 10 mM NaBr, but (A) and (B) display G4/DNA and CTAB/DNA complexes, respectively, after being transferred to the tRB used for *in vitro* transcription/translation experiments. (C) and (D) are the reference samples in the 10 mM NaBr solution used for DNA compaction. Scale bars are 100 nm, the arrow indicates frost (artifact), the white stars indicate the carbon film and the black star shows a fracture of the vitreous film.

However these structural studies do not allow us to directly quantify the interaction, i.e. the number of dendrimers that bind to each DNA molecule. Here we therefore use steady state fluorescence spectroscopy to quantify the amount of free DNA at varying *r*_charge_, Figure 1. The fluorophore GelStar is unable to bind to DNA in its compacted form and the emitted fluorescence is linearly dependent on the “free” DNA concentration (in the absence of compacting agent). We have previously used this method to estimate the compaction of salmon sperm DNA (2000 bp) by G4 dendrimers [55]. The same concept was earlier developed by Chen et al who extensively studied binding of the fluorophore ethidium bromide for three different dendrimer/DNA ratios [58]. Based on their data they could extract information on both the binding constant of the fluorophore, but also estimate the number of dendrimers per DNA molecule. The aim with the present study is to reveal the amount of “free” DNA as a function of the amount of added compacting agent. Figure 1A shows the relative fluorescence intensity normalized with intensity without the compacting agent as a function of *r*_charge_ values for linearized plasmid DNA of 4331 bp in the presence of CTAB surfactant or G4 dendrimer. The fluorescence emission intensity is shown to gradually decrease with increasing *r*_charge_ values. This indicates that the amount of free DNA decreases for increased concentrations of compacting agent. For the G4/DNA system, the amount of free DNA decreases (about) linearly with the amount of added G4, till nearly all DNA is compacted at *r*_charge_ ≈1. Assuming that all dendrimers added bind to DNA for *r*_charge_ <1 [55], the mean number of G4 dendrimers binding per DNA chain can be estimated based on the known added amount of dendrimers and the fraction of compacted DNA observed in the fluorescence measurements. Figure 1B shows the calculated number of G4 per compacted DNA as a function of *r*_charge_. For *r*_charge_ values up to 1.1, the number of dendrimers per DNA molecule is almost constant and equal to 140. For larger *r*_charge_ values, when no more free DNA is present in solution (Figure 1A), the calculated number of G4 dendrimers per compacted DNA molecule increases. Previous results recorded for 2000 bp DNA at similar *r*_charge_ values, showed that 35 dendrimers bind per DNA molecule. We can therefore conclude that the 4331 bp DNA molecule, studied here, appears to bind a proportionally higher fraction of G4 dendrimers. The cryo-TEM images in Figure 2 confirm the formation of compact aggregates of DNA/G4 dendrimer complexes at high *r_charge_* of 1.5. Theoretical prediction shows that the number of dendrimers per DNA chain is dependent on the DNA length, the penetrability (or softness) of the dendrimer and the stiffness of the DNA chain [59]. Better agreement was found between the model and the experimental data for the 4331 bp DNA than for the 2000 bp DNA, but based on this relatively simple model we could not directly determine the reason for the observed DNA length dependence. However, an important conclusion from the analytical model study was that the radius of the dendrimer in the dendrimer/DNA complex has to be smaller than that of the free dendrimer. This means that the dendrimer has to contract or the DNA has to penetrate into the dendrimer upon DNA binding and/or compaction in order to accommodate the number of dendrimers necessary to sufficiently neutralise the DNA charge.

The number of CTAB micelles in the complexes is not as straightforward to calculate since the CTAB aggregation number on DNA is unknown. Furthermore, in contrast to the G4/DNA system, the fluorescence emission intensity for the CTAB/DNA system never reaches zero, even at relatively high surfactant concentrations. The emission intensity does not start to decrease at the same low *r*_charge_ values as for G4 dendrimers (<0.2). At *r*_charge_ ≈1 where *I*_x_/*I*_max_ ≈0 for G4, the corresponding intensity value for the CTAB system is still nearly 1. The results reported here for the CTAB/DNA system are in good qualitative agreement with previous studies using other fluorophores, such as Ethidium Bromide (EtBr) [60] and YOYO [61]. Furthermore, a decrease in fluorescence intensity is observed at *r*_charge_  = 3 and for *r*_charge_ >10, the fluorescence intensity does not decrease much further and a second plateau is reached. This shows that it is not possible to completely hinder the interaction of DNA with GelStar using surfactants, which agrees with fluorescence microscopy observations [62]. One could imagine that CTAB micelles within a complex may also partly include some GelStar. However this is contradicted by the observation that GelStar does not produce fluorescence with the surfactant in the absence of DNA.

Data from gel electrophoresis experiments, shown in Figure 1C and 1D for G4 and CTAB, respectively, confirm the results in Figure 1A. First a high intensity of the gel electrophoresis band corresponding to free (naked) DNA can be observed in the presence of increasing amounts of the G4 dendrimer at low *r*_charge_ values (Figure 1C). At intermediate *r*_charge_ values (≤1), coexistence of free and complexed DNA is observed. Finally, the bands corresponding to complexed DNA, that is, those remaining in the well or running more slowly, are more intense. This shows that the G4/DNA complexes are less negatively charged than free DNA or even neutral. These data also agree well with the study by Kukowska-Latallo et al. although their study mainly focused on conditions where *r*_charge_ >1 [63]. It should here be noted that for *r*_charge_ >1, the complexes might also precipitate to some extent. For CTAB the neutralization of the DNA charges is observed at a higher charge ratio (Figure 1D) than with G4 dendrimers (Figure 1C) and the DNA band is not affected until *r*_charge_ ≥2. This is in good agreement with previously published DNA-surfactant phase maps [56], which show that for very low concentrations of DNA (water-rich corner) the concentration of CTAB required to induce precipitation is larger than *r*_charge_  = 1. This is a typical behavior for polyelectrolyte-oppositely charged surfactant systems [64], [65]. It is interesting to note that the CTAB/DNA complexes at *r*_charge_ >1 still migrate in the gel in the same direction as DNA, which shows that they still carry some negative charges. The presence of charge increases the aqueous solubility of the complexes and consequently no macroscopic phase separation is visible.

As for the G4/DNA system, free DNA is observed together with CTAB/DNA complexes, which are similar in size to the globular complexes containing G4 (Figure 2C and D). Using cryo-TEM it was also observed that the electron beam more easily burned DNA within CTAB/DNA complexes compared to within G4/DNA complexes. This indicates that G4 is more efficient in compacting the DNA molecule, which agrees with the fluorescence spectroscopy data that shows that CTAB does not totally exclude GelStar from DNA, even at high *r*_charge_ values (Figure 1A).

We speculate that the self-assembly character of the CTAB/DNA complex results in a more flexible structure that allows for a more efficient packing in the complex core than in its exterior. The high DNA stiffness and the fact that CTAB forms rod-like micelles in the vicinity of DNA, which can be packed in a hexagonal array, limits the number of DNA segments to be accommodated in the core [66]. Protruding DNA segments, to which less or no CTAB binds, could explain why CTAB induces complexes with a negative net-charge and also why they bind GelStar at higher *r*_charge_ values and are observed to be more susceptible to beam damage using cryo-TEM.

### DNA compaction reduces *in vitro* gene expression

**Figure 3:**
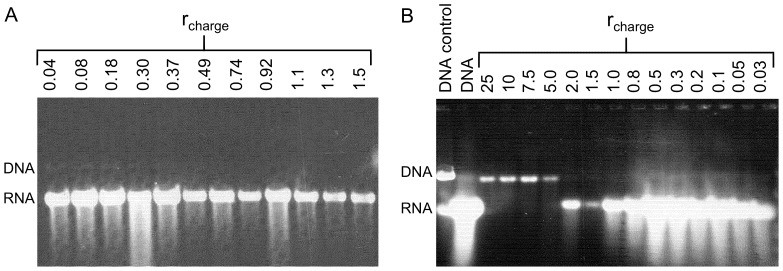
Luciferase gene expression and DNA accessibility as a function of *r*_charge_ using pre-casted RNA gels. (A) G4 dendrimers and (B) CTAB. The synthesized amounts of RNA are displayed and samples were not pretreated with Dnase I. References are displayed in B where lane 1 shows the sample consisting only of DNA and without any compacting agent or transcriptional activity. Lane 2 shows the control sample containing DNA and the *in vitro* transcription mixture in the absence of compacting agents. Gels were post-stained using GelStar.

The key objective of the present study is to reveal the impact of DNA compaction, induced by either G4 dendrimers or CTAB, on the luciferase gene expression. As described in the experimental section, DNA and compacting agents were mixed in aqueous solutions of 10 mM NaBr and aliquots of the samples were transferred to solutions containing tRB. The effect of this change of buffer will be discussed further below. From the electrophoresis gels in Figure 3 we can estimate the amounts and monodispersity of RNA produced during *in vitro* transcription of DNA as a function of *r*_charge_ values for G4 (A) and CTAB (B). The amount of RNA generated is reduced with increasing concentrations of compacting agents, as indicated by the decreased intensity of the RNA bands at high *r*_charge_ values. However, also at the maximum concentration of G4 dendrimers used in this study (*r*_charge_  = 1.5), the production of RNA is not completely suppressed (Figure 3A), despite the formation of stoichiometric G4/DNA complexes as shown by the zero electrophoretic mobility (Figure 1C). For the CTAB/DNA system, however, no RNA could be detected at *r*_charge_ ≥5.0 (Figure 3B), which indicates inhibition of transcription. This *r*_charge_ value corresponds to the *r*_charge_ when DNA retention is observed in the gel electrophoresis experiment (Figure 1D), even though an even higher *r*_charge_ is needed for the complex to become completely immobilized. We also note that DNA bands are observed at high CTAB concentration when no RNA synthesis could be detected. This is not observed for G4 and is consistent with the fact that it is not possible to completely hinder the interaction of DNA with GelStar using surfactants. This suggests the presence of free DNA even at high CTAB concentrations, at least when the samples are subject to an electric field. Although the purpose with the electrophoresis study was to reveal the effect of CTAB on the RNA production we note that we do not observe any DNA bands at lower surfactant concentration even if RNA synthesis is observed. It is obvious that the gel is overloaded when it comes to RNA and it can therefore be difficult to observe DNA when high amounts of RNA are produced. Here we note that the GelStar bind to both DNA and RNA for the binding of GelStar.

**Figure 4:**
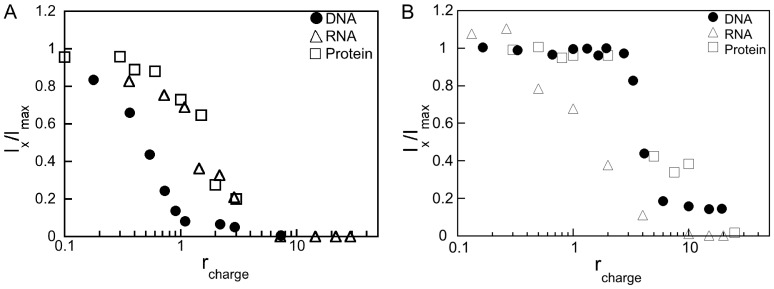
Inhibition of *in vitro* transcription and translation as a consequence of DNA compaction. (A) G4 dendrimers and (B) CTAB. The (SYTO RNASelect) fluorescence intensity (▵) corresponds to the produced amount of RNA and the GelStar exclusion data from Figure 1A (•) is added as a reference. The chemiluminescence intensities corresponding to the produced amount of luciferase (□) for both the G4/DNA and the CTAB/DNA systems are also shown. The concentration of linearized plasmid DNA is 2 μg mL^−1^ in the GelStar exclusion experiments and 10 μg mL^−1^ in the *in vitro* transcription and translation experiments. Data are normalized to the amounts produced in the samples only containing DNA (without compacting agent).

It is clear that it is difficult to quantify the amount of RNA produced from the gel electrophoresis experiments, e.g. less band intensity observed in the lane of *r_charge_*  = 1.5 than for *r_charge_*  = 2.0 in Figure 3B. We therefore used steady state fluorescence spectroscopy employing the SYTO RNASelect stain (Figure 4A and B for G4 and CTAB, respectively) to determine the amount of RNA produced when using the *in vitro* transcription assay. In this assay the intensity of RNA signals (*I*_x_) normalized with the maximal intensity recorded for the sample without any compacting agent (*r*_charge_  = 0, *I*_max_), is determined as a function of *r*_charge_.

For both systems we also tested for translation capability of the produced RNA that is, the production of luciferase by coupling *in vitro* transcription to *in vitro* translation. The amount of luciferase was quantified by determining the luciferase enzyme activity. The dye exclusion from complexed DNA (Figure 1A), that is, the decrease of free DNA, is included in Figure 4 as a reference. For the G4/DNA system, even small amounts of G4 result in a decrease of the luciferase activity (Figure 4A). This corresponds to a decrease in the RNA production and it is noteworthy that *r*_charge_ values have to be ≥1.5 to significantly reduce the genetic activity, in agreement with Figure 3A. This is significantly higher than the *r*_charge_ values when no free DNA could be detected based on the absence of GelStar fluorescence displayed in Figure 1A and C. DNA is, however, still transcribed to some degree by the T7 RNA polymerase even if all DNA molecules should be compacted. This either indicates that T7 RNA polymerase can function on compacted DNA or that there is a sufficient amount of free DNA, not detectable by fluorescence spectroscopy, which can be transcribed. However, for higher values of *r*_charge_ (*r*_charge_ ≥7) and for the incubation time used in this study (2 h), the synthesis of RNA appeared to be suppressed. It is clear that the most drastic decrease in RNA production and Luciferase activity occurs between *r*_charge_  = 1 and *r*_charge_  = 2, where *I_x_/I_max_* drops by a factor of 3. Here it should be noted that Megascript kit (Promega) was used to produce the RNA for analyses, whereas a coupled cell free transcription-translation system (TNT Coupled Reticulocyte Lysate System, Promega L4610) was used to synthesize luciferase. In general the trends in terms of change in *I_x_/I_max_* versus *r*_charge_ coincides, but for for *r*_charge_  = 1.5 where the largest changes in *I_x_/I_max_* is expected the values corresponding to RNA production is significantly lower than the corresponding luciferase activity. This is most likely dependent on differences in performance of the two systems, which show up when the largest changes in RNA production and Luciferase activity are expected. In conclusion the strong correlation between the RNA production and translation into Luciferase activity makes it unlikely that G4 on its own significantly affect the luciferase activity under the conditions used in this study.

The results for the CTAB/DNA system are also consistent with the hypothesis that the level of *in vitro* transcription depends on the concentration of compacting agent as the amount of RNA produced decreased with increasing CTAB concentration (Figure 4B). Furthermore, no detectable amount of RNA is produced at *r*_charge_ ≥10 even though DNA migration was still observed in gel electrophoresis (Figure 1D). For this value of *r*_charge_, binding of the GelStar fluorophore to DNA could still be detected and so the *r*_charge_ at which gene expression was suppressed was lower than what could be expected from the dye exclusion assay (Figure 1B). It is interesting to note that the *in vitro* transcription data in Figure 4A and B, that is, the *I*_x_/*I*_max_ corresponding to the (normalized) amount of generated RNA, is determined by the *r*_charge_ values rather than the type of compacting agent used. However, here we note that the luciferase production when using CTAB as compaction agent is strongly reduced at higher surfactant concentration, i.e. at the same *r*_charge_ values as when the GelStar fluorophore binding to DNA is strongly reduced. The reason why the RNA production is switched off at much lower *r*_charge_ values than luciferase activity in this case are likely due to differences in the assays. In fact also for the dendrimers we found the production of RNA decreases more drastically than the decrease in luciferase. The presence of the surfactant might interfere with the system and thus the complete “switching off” of the protein production occurs for much larger concentrations of CTAB in the case of the transcription-translation system than Megascript systems.

To summarize, G4 data in Figure 4 show that the compaction of DNA, in aqueous 10 mM NaBr solutions (based on the determination of free DNA from GelStar fluorescence intensity), occurs at a lower value of *r*_charge_ compared to the inhibition of *in vitro* transcription/translation which is taking place in aqueous solutions containing the necessary tRB. However, when CTAB is used as a compacting agent the opposite is observed, that is, a higher value of *r*_charge_ is required for full DNA compaction than for transcription inhibition, see Figure 4B. Here it should be pointed out that for CTAB the inhibition of *in vitro* luciferase synthesis coincides with the compaction of the DNA. The reason for this discrepancy is not entirely clear, but is likely to be due to differences in the assays. In spite of these discrepancies we can conlude that both G4 and CTAB can be used to almost completely shut off transcripition as well as transcription/translation as showed by using two different types of assays. Furthermore we can link this to the compaction of the DNA.

### Influence of the tRB on the structure of DNA and DNA-complexes

The tRB required for gene expression contains di- and trivalent species (MgCl_2_ and spermidine), which are known to promote DNA compaction (see the full list of components of the tRB in the experimental section). These compounds are important for transcriptional activity but might, on the other hand, affect the performance of the compacting agent. Raspaud *et al.* have shown that mononucleosomal DNA of 146 bp can form precipitates that are liquid crystalline in the presence of spermidine (+3), and spermine (+4) [67], which are common components in reaction buffers required for gene expression. Although they used a significantly shorter DNA than in the present study, we cannot rule out that the tRB affects the DNA compaction process.

**Figure 5:**
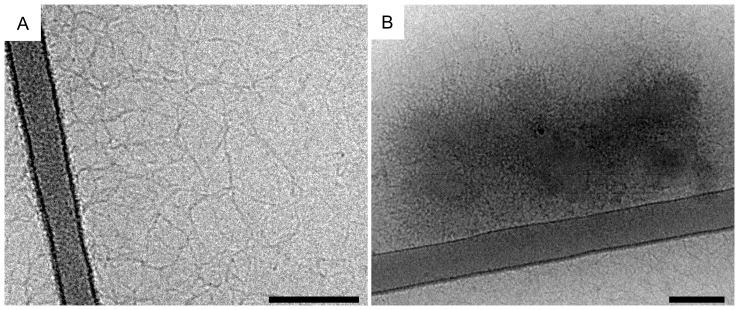
Cryo-TEM of DNA dissolved in the tRB used for *in vitro* transcription and translation experiments. Coexisting domains of free DNA (A) and tightly packed clusters of DNA (B) are shown. Scale bars are 100 nm and the DNA concentration is 0.1 mg mL^−1^.

In order to elucidate relevant changes in the nanostructure of the complexes induced by the addition of the tRB, we performed cryo-TEM. Figure 5 shows the corresponding micrographs for DNA in the absence of compacting agents dissolved in the tRB. DNA is found both as individual molecules (A) and as tightly packed clusters (B). The presence of the clusters show that the DNA possibly can be associated in the same type of liquid crystalline domains as observed by Raspaud *et al.*, even though the DNA used in the present study probably is too long to form well-defined liquid crystalline domains. No compact DNA clusters have previously been observed in 10 mM NaBr solutions [27]. We therefore conclude that the presence of the tRB changes the DNA morphology and promotes a more compact DNA structure.

Cryo-TEM was also performed on G4/DNA and CTAB/DNA complexes. Figure 2 displays the micrographs for G4/DNA of *r*_charge_  = 0.5 (A) and CTAB/DNA of *r*_charge_  = 7.5 (B), which were prepared in 10 mM NaBr, after being transferred to tRB. Figure 2C and D display the reference samples in the 10 mM NaBr solution used for DNA compaction and clear differences between complexes in the absence and presence of tRB is observed. For both G4/DNA and CTAB/DNA systems the complexes are larger in tRB than in 10 mM NaBr. This is particularly obvious when G4 is used as the compacting agent for which aggregates cover nearly the entire TEM grid. It is difficult to directly relate the morphology to the level of genetic expression but it is clear that the presence of large G4/DNA complexes (Figure 2A) does not prevent genetic activity (see Figure 3 and 4). The CTAB/DNA system also shows some aggregation but to a lower extent compared to G4/DNA, probably due to the fact that individual CTAB/DNA complexes are more negatively charged (Figure 2D).

**Figure 6:**
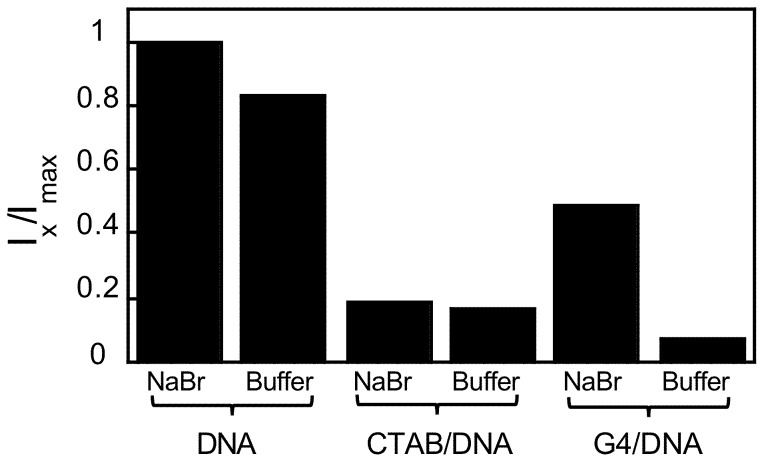
The solvent effect on DNA compaction using steady state fluorescence spectroscopy. Columns marked as NaBr correspond to samples in aqueous solutions of 10*in vitro* gene expression kit. Data are normalized to the emitted intensity in the sample only containing DNA (without compacting agent) in 10 mM NaBr solution (1^st^ column). The *r*_charge_ values reported for the samples containing compacted DNA are 7.5 for CTAB/DNA and 0.5 for G4/DNA. The DNA concentration is 2 μg mL^−1^ and the dye used is GelStar.

To gain further insight into how the bulk salt composition changes the morphology of the complexes, steady state fluorescence spectroscopy data on the amount of free DNA (available to bind GelStar) was obtained in tRB and compared to data in aqueous solutions of 10 mM NaBr. Figure 6 shows the GelStar fluorescence intensity obtained for free DNA (*r*_charge_  = 0), CTAB/DNA of *r*_charge_  = 7.5 and G4/DNA of *r*_charge_  = 0.5, relative to free DNA in a 10 mM NaBr solution. The amount of free DNA, available to bind GelStar, is shown to decrease when placed in the tRB. This is consistent with Figure 5, which displays clustering of DNA in the presence of the tRB not observed in 10 mM NaBr [27]. Only a minor effect on the free DNA concentration, as monitored by the bind GelStar binding, is observed for the CTAB/DNA samples with tRB as solvent compared to using 10 mM NaBr solution. A significant effect is, however, observed for the G4/DNA samples, where the fluorescence intensity, that is, the capability of GelStar to bind DNA, decreases to a minimum when the complexes are exposed to the tRB. These results agree with Figure 2 where the tRB induces large G4/DNA aggregates, but only slightly larger CTAB/DNA complexes, compared to those in 10 mM NaBr solutions. These results show that the tRB promotes compaction in the presence of G4 dendrimers, but on its own it only reduces the availability of DNA for GelStar binding by about 15% compared to 10 mM NaBr.

### Releasing DNA from complexes so that transcription is resumed

**Figure 7:**
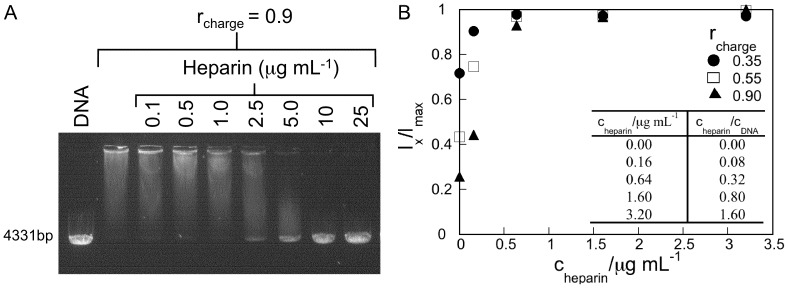
Decompaction of G4/DNA complexes using the polysaccharide heparin. (A) GelStar-stained gel showing the degree of dissociation of G4/DNA complexes (*r*_charge_  = 0.90) for the indicated concentrations of heparin. The 1^st^ lane shows the linearized plasmid DNA only (25 μg mL^−1^, control). (B) GelStar fluorescence measured by steady state fluorescence spectroscopy as a function of heparin concentration. Measurements are performed for DNA (2 μg mL^−1^) with complexes of *r*_charge_  = 0.35 (•), 0.55 (□) and 0.90 (▵). The intensity is normalized to that of free DNA (in the absence of G4) and error bars are smaller or equal to the marker size. Inset shows the concentration ratios of heparin and DNA.

We have so far demonstrated that we are able to largely reduce *in vitro* gene expression from DNA by forming complexes with CTAB and G4 dendrimers. One important aspect for applications is whether DNA can be released from the complex or not, which also provides insight on the strength of the interaction between the compacting agent and DNA. The possibility to disrupt dendrimer/DNA complexes formed at high *r*_charge_ values by the anionic surfactant SDS has previously been reported [28]. However, the G4/DNA complex of *r*_charge_ <1 in this study could not be dissociated in the presence of either anionic (SDS) nor nonionic (pentaethyleneglycol monododecyl ether (C_12_E_5_)) surfactants even at high surfactant concentration, as observed by gel electrophoresis (data not shown). Instead we used the negatively charged polysaccharide, heparin, which is common in biological systems. It should here be noted that heparin has been suggested to have a range of other biological functions beyond its anticoagulant activity [68]. Apart from being present in the extracellular matrix of the biological system it has also come into large clinical use [69]. Heparin has previously been found to mediate DNA release from, for example, other polyelectrolytes as well as cationic liposomes [70], [71]. Indeed we obtained efficient DNA release from G4, as demonstrated by gel electrophoresis and steady state fluorescence spectroscopy, Figure 7. Figure 7A shows the results from a gel electrophoresis experiment where the gel was loaded 30 min after addition of heparin to G4/DNA of *r*_charge_  = 0.90. The amount of DNA released from G4 increases with increasing heparin concentration and for 10 μg mL^−1^ all DNA is released. Note that this value is lower than the used DNA concentration of 25 μg mL^−1^. An identical gel electrophoresis image was obtained after an incubation time with heparin of 24 h (data not shown) and we conclude that decompaction and release of DNA is completed within 30 min. Fluorescence spectroscopy was also performed to quantify the G4/DNA (2 μg mL^−1^ DNA) complex dissociation using heparin, Figure 7B. The amount of heparin needed for decompaction depends on the dendrimer concentration and for *r*_charge_  = 0.90, the results reveal that nearly all DNA is released with ∼1.6 μg mL^−1^ heparin, which agrees well with the data in Figure 7A.

Heparin is a highly charged polymer that is likely to compete for the dendrimer cationic groups with the also highly charged DNA. If we compare the charge density of the two polymers we find that for heparin the charge density is ∼1e^−^ per 0.47 nm [72], whereas DNA has a linear charge density of 1e^−^ per 0.17 nm [73]. Based on these data heparin would not be expected to expel dendrimers from DNA. However, the persistence length of heparin is 4.5 nm [72], whereas DNA is known to be a semi-flexible chain with a persistence length of 50 nm [73]. It is therefore likely that the ability for heparin to compete for the dendrimer charges is due to its higher flexibility in combination with its high charge and charge density.

**Figure 8:**
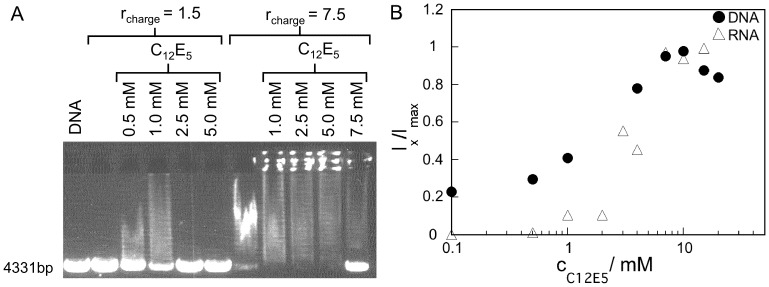
Dissociation of CTAB/DNA complexes using C_12_E_5_. (A) Gel stained with EtBr where lane 1 shows the linearized plasmid DNA only (control). The CTAB/DNA samples loaded onto the other lanes are of *r*_charge_  = 1.5 (0.11 mM) and 7.5 (0.57 mM), and were treated with the indicated amounts of C_12_E_5_. The DNA concentration is 25 μg mL^−1^ (75.8 μM). (B) Fluorescence intensities are measured by fluorescence spectroscopy as a function of C_12_E_5_ concentration. The GelStar intensities (•) reflects the amount of free DNA and the SYTO RNASelect intensity (▵) reflects the amount of RNA produced by *in vitro* transcription. Error bars are smaller or equal to the size of the markers. The concentrations of DNA and CTAB are 2 μg mL^−1^ and 60.6 μM, respectively, (*r*_charge_  = 10) in the GelStar exclusion experiments and 10 μg mL^−1^ and 0.30 mM, respectively, (*r*_charge_  = 10) in the *in vitro* transcription experiments. The intensity was normalized to the sample only containing DNA (in the absence of CTAB).

We have, additionally, addressed the route of CTAB/DNA complex dissociation (Figure 8). For a system controlled by surfactant assembly it is natural to think of a self-assembly route also for the decompaction and release of DNA from the complex. We observed that, in contrast to dendrimers, anionic and even nonionic surfactants are efficient in releasing DNA [40], [74], [75]. Figure 8A displays gel electrophoresis data where the nonionic surfactant C_12_E_5_ is used to release the DNA from the CTAB/DNA complex. The experiments were performed with two different CTAB concentrations and the results show that at higher CTAB concentrations an increased concentration of non-ionic surfactant is required for complex dissociation. This is probably because complex dissociation requires the formation of mixed surfactant micelles and so the more CTAB that is present in solution, the more C_12_E_5_ will be required to compete for the binding of CTAB with the DNA molecules. We note that for the neat systems, the critical micellar concentration (*cmc*) of CTAB and C_12_E_5_ in aqueous solutions at 25°C are 0.92 mM and 65 μM respectively [76]. Thus, C_12_E_5_ is expected to have a higher tendency to form micelles (lower *cmc*) than CTAB and, although we do not know the *cmc* of the mixed system, we expect it to have an even lower value than that of C_12_E_5_, since the formation of mixed micelles efficiently reduces the effective charge of the ionic surfactant micelle as well as the steric repulsions between the C_12_E_5_ surfactant headgroups. The dissociation of the CTAB/DNA complexes by C_12_E_5_ was also investigated by fluorescence spectroscopy. Figure 8B shows the fluorescence intensity value obtained for each of the samples containing CTAB (0.75 mM, *r*_charge_  = 10) as a function of C_12_E_5_ concentration. The DNA was stained with GelStar and the signal was normalized as described earlier. The data show that the fluorescence intensity increases with increasing concentrations of non-ionic surfactant and at a C_12_E_5_ concentration of ∼7.0 mM (global composition of ∼10 C_12_E_5_ molecules for each CTAB molecule), the fluorescence intensity is completely restored and DNA is again accessible to GelStar binding. The decrease in fluorescence intensity at high concentrations of C_12_E_5_ (*r*_charge_ >10) could be due to interactions of the fluorophore with the mixed micelles. As previously discussed, it is possible that GelStar is solubilized in the interior of the surfactant micelles, as has been observed for other nucleic acid stains [75]. In this case less fluorophore molecules would be available for DNA binding, leading to a decrease in the fluorescence intensity. In addition, Figure 8B shows the transcriptional competence of the DNA as a function of C_12_E_5_ concentration. Increased amounts of C_12_E_5_ lead to increased amounts of synthesized RNA and thus, the accessibility of the DNA for transcription is restored. Together, the results show that it is possible to regulate the transcription of DNA by changing the composition of the surfactant mixtures. The inhibition of transcription observed in the CTAB/DNA systems is therefore not to be ascribed to the presence of the cationic surfactant per se, but to the complexation and compaction of the DNA, that is, a decrease of concentration of DNA in the semi-flexible coil state.

### Compacted DNA is protected against degradation

**Figure 9:**
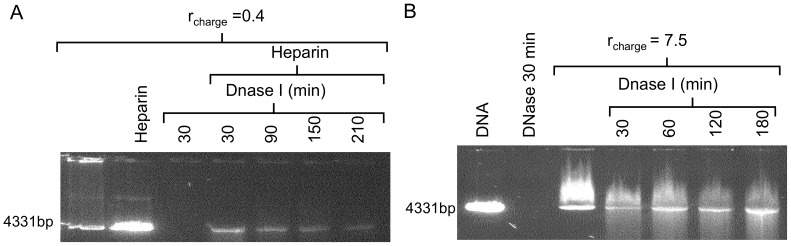
Protection of DNA against Dnase I digestion using gels stained with GelStar. (A) G4/DNA complexes with *r*_charge_  = 0.4 are used and the untreated complex is loaded on lane 1. The 2^nd^ lane displays the dissociated complex after treatment with 10 μg mL^−1^ heparin for 30 min. All other samples (lanes 3–7) are treated with 1 unit of Dnase I for the indicated time periods. To the samples in lanes 4–7, heparin was added after the Dnase I enzyme was heat inactivated. (B) Linearized plasmid DNA only is loaded onto lane 1 and the sample loaded onto lane 2 contains DNA, treated with Dnase I for 30 min. Samples loaded onto lanes 3–7 contain DNA and CTAB (*r*_charge_  = 7.5). Samples loaded onto lanes 4–7 are treated with Dnase I for the time periods indicated.

An important reason to compact DNA in semi-biotic systems is to protect it from degradation. We therefore compared the ability for the compacting agents to form a DNA-containing complex in which the DNA is protected from being digested by Dnase I. For this purpose, samples containing compacted DNA were incubated for various time periods with Dnase I and the samples were analysed with gel electrophoresis. Figure 9 shows the results when DNA is protected by G4 for *r*_charge_  = 0.4 (A) and CTAB for *r*_charge_  = 7.5 (B). In Figure 9A, lane 1 displays the migration of the untreated G4/DNA sample and as for *r*_charge_  = 0.4 not all DNA is compacted we observe a band corresponding to free DNA. The release of DNA by heparin is shown in lane 2 as an increase in intensity of the band corresponding to free DNA. When no heparin is added to the complexes after incubation with Dnase I, no DNA band is detected, even if the samples are incubated with Dnase I for only 30 min (lane 3). It appears as all DNA has been completely degraded. However, when heparin is added to samples that have been incubated with Dnase I, a band corresponding to free DNA is now obtained even after incubation of the complexes with Dnase I for 30, 90, 150 and up to 210 min. Due to the cooperative manner of DNA compaction, the fraction of DNA included in the complex will be protected against degradation, but the fraction of free DNA (at *r*_charge_ <1) will be degraded. The results agree with the results from previous studies that show that at r_charge_ >1, dendrimers protect DNA against nuclease activity [28]. It is also observed that when the G4/DNA complexes are incubated for longer time using Dnase I, the intensity of the DNA band decreases (Figure 9A, lanes 4–7). These results show that the DNA is not completely protected, but that the rate of degradation is strongly decreased in the presence of dendrimers. Increased accessibility might be due to either a reorganization of the complex during digestion or to the penetration of Dnase I into the complex. Free DNA is completely degraded in less than 20 min after enzyme addition (data not shown) and after 30 minutes no DNA is observed (Figure 9B, lane 2), Dnase I is expected to initially act on all DNA segments protruding out from the complexes into the solution, forming loops and tails. This provides also a possible explanation to why no DNA is detected in the gels prior to the heparin treatment. If the free DNA sequences, which are accessible to Dnase I, are digested, only a compact complex remains to which no or a negligible GelStar amount can bind.

**Figure 10:**
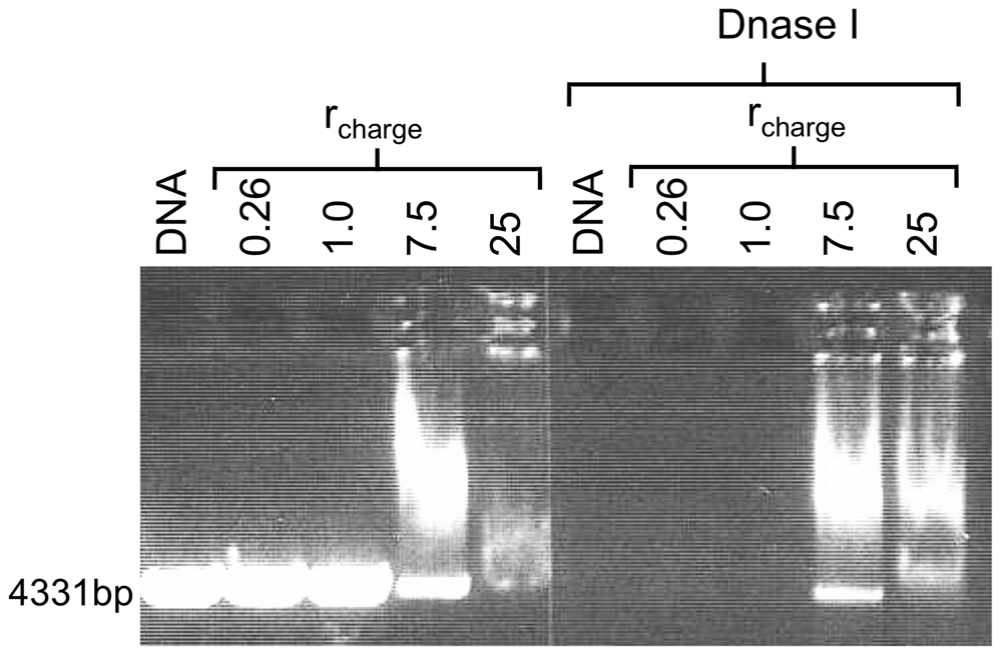
Protection of DNA against Dnase I digestion by CTAB using a gel stained with EtBr. A gel electrophoresis gel where samples in lanes 1 and 6 contain linearized plasmid DNA only (control). The remaining lanes contain CTAB/DNA of the *r*_charge_ values indicated. The samples loaded onto lanes 6–10 were incubated with Dnase I for 20 min following DNA condensation. At *r*_charge_ ≤1.0, the DNA is completely degraded. At higher concentrations of CTAB, DNA degradation is inhibited.

Figure 9B shows the efficient protection of DNA against Dnase I digestion offered by CTAB for at least 3 h. However, DNA degradation occurs also here to some degree as indicated by the smear of shorter DNA fragments below the main band of 4331 bp. It merits mentioning that *in vitro* transcription (Figure 3–4) and DNA degradation (Figure 9) were effectively inhibited at similar *r*_charge_ values. Thus both Dnase and polymerase have difficulties in reaching and progressing along the DNA molecule, most likely due to the fact that most of the DNA molecules are compacted. In addition, Figure 10 shows that DNA is completely digested at low concentration of CTAB, as indicated by the absence of bands for *r*_charge_ <7.5. There are no predominant DNA fragments with specific sizes for either G4 or CTAB, which indicate that the dendrimer or surfactant complexes do not bind to specific locations on the DNA molecule [28].

## Conclusion

One of the key objectives was to compare the effect of a compacting agent that is mulitvalent (G4 dendrimer) with a monovalent surfactant, CTAB, that assembles into a multivalent one, i.e. a CTAB micelle. We have shown using fluorescence spectroscopy and electrophoresis that dendrimers are more efficient in retarding the DNA electrophoretic mobility than CTAB. While DNA does become fully inaccessible to fluorophore binding when complexed with high concentrations of dendrimers, this does not occur when DNA is complexed with CTAB. Thus it seems that the highly charged dendrimers induce a higher overall degree of DNA compaction. G4 dendrimers display a (maximal) charge of +64 whereas CTAB are cationic surfactants with one positive charge in the headgroup, which are self-assembled into rod-like micelles in the vicinity of DNA, forming more or less ordered hexagonal surfactant/DNA structures [66]. For DNA and CTAB or G4, respectively, the presence of the multivalent tRB induced aggregation of the complexes. It seems, however, that the formation of larger aggregates reduced the binding of GelStar. In this respect the morphology of CTAB/DNA complexes was found to be less sensitive to changes of buffer conditions compared to G4/DNA. We propose that this is due to the higher net charge of the CTAB/DNA complexes, compared to the neutral G4/DNA complexes, likely to give higher stability against the formation of large aggregates.

*So how are these differences between G4 and CTAB DNA-complexes reflected in the ability to regulate transcription*? *In vitro* cell-free gene transcription appeared to be reduced at *r*_charge_ values that are rather similar independent on whether DNA is compacted with G4 or with CTAB. However, when it comes to the coupled transcription/translation for the synthesis of luciferase it seem that for CTAB the inhibition of *in vitro* luciferase synthesis coincides with the compaction of the DNA at higher *r*_charge_ than transcription inhibition. It is interesting to note that the accessibility for GelStar is greater for CTAB/DNA than G4/DNA complexes under conditions where the reduction in transcription and degradation is the same. The main conclusion from this work is that both G4 and CTAB can be used to almost completely shut off transcripition as well as transcription/translation and this can be linked to the compaction of the DNA.

*Protection of the complexed DNA against digestion was studied using Dnase I and both compacting agents offered protection during similar time periods.* Total protection was, however, not achieved and some degradation occurred with time. A higher r_charge_ is required in the case of CTAB to achieve efficient protection against degradation compared to G4. No enrichment of DNA fragments with specific sizes was observed in the fractions of degraded DNA, which suggests that neither CTAB nor G4 bind in an ordered fashion to the DNA molecules [28]. Another not mutually exclusive possibility would be that the CTAB complexes or G4 dendrimers slide along the DNA molecules.

*One objective was to exploit the possibilities to reverse the compaction for the two types of compacting agents and restore the transcription capability.* Dissociation of the G4/DNA and CTAB/DNA complexes was efficiently achieved using heparin and non-ionic surfactants, respectively, and gene expression was successfully resumed for the later system. Here we also found that it was impossible to dissociate the G4/DNA complex with neither an anionic nor nonionic surfactants.
